# Relations between the Food and Flesh of Animals

**Published:** 1836

**Authors:** 


### WESTERN JOURNAL

OF THE

### MEDICAL AND PHYSICAL SCIENCES:

**For January, February and March, 1836.**

### ESSAYS AND CASES.

### Art. I.—Relations between the Food and Flesh of Animals.

*Facts relative to the Influence of Variations of Food on the qualities of the Flesh and Fluids of Animals.* By the Editor.

During the past winter, the discussions in the Cincinnati Medical Society, took the course of solidism and humoralism, and excited so much interest, that the Editor has resolved on publishing in the Journal, one of his lectures on the *Materia Alimentaria*, delivered in Transylvania University, November, 1823. Being shortly afterwards transferred from the chair of *Materia Medica* to the *Practice of Medicine*, he did not enlarge and perfect this lecture; and does not now regard it as of deep interest, for many of the facts will be familiar to most of his readers. Still, it may, perhaps, be found equal in point of utility, to many of the matters which find a place in the Journals of the day.

It is well known, that the flesh of animals of different species, varies exceedingly in its qualities, and no doubt their blood and other fluids differ in an equal degree. These variations among different tribes, compared with each other, are in harmony with those anatomical and physiological differences, which constitute them into distinct species. It is not my design, to inquire into the influence which the diet of each species exerts on the qualities of its flesh, compared with other animals, but into the effects on individuals, compared with other individuals of the same species, of departures from their natural aliment. These effects are unquestionable, but their limits are not very extended; for there is a *vis conservatrix naturæ*, in every living system, which is directed to the maintenance of a uniform condition. How far it succeeds, under great variations of diet, drink, and condiments, can only be determined by observation and experiment. Relying then upon facts, rather than *a priori* reasonings, I shall proceed to enumerate such as have presented themselves to me in a moderate course of inquiry, dividing them into several distinct heads.

I. Of the modifications which have been observed in the fluids, from subjecting animals to the ingestion of particular substances.

1. *Of the Chyle.*—Dr. Marcet analyzed the chyle of dogs subjected, successively, to a diet purely vegetable and purely animal. The chyle from vegetables kept for several weeks without putrefying—and contained a great deal of carbon. The chyle from animal food putrefied in a few days; it had a layer of oil swimming on it like cream which the other had not; it abounded in ammonia to a much greater degree than the other, and contained but a third part as much carbon. (Orfila. Chem. Anim. vol. 2, p. 403.)

Dr. Johnson (Animal Chemistry, vol. 1, p. 212) observes—"A great many substances enter the lacteals along with the chyle, even solids reduced to a fine powder. When Indigo has been thrown into the intestine of a sheep, Dr. Fordyce has seen the chyle rendered quite blue; yet indigo is not soluble in water, but is a solid reduced to fine powder. Musk likewise gets into the chyle, giving it a strong smell, and a great variety of other substances of various colors, various tastes, and various smells, each of them giving color, taste, or smell to the chyle."

Magendie (Phys'y. p. 378) asserts that the chyle of dogs fed exclusively on gum or sugar, becomes transparent and watery.

2. *Of the Milk.*—Modifications of the milk of woman and the mammaliæ, have been observed to follow the use of certain kinds of aliments. In reference to woman, it has been said, that, disordering her health, they disturb the mammary secretion; and thus, render her milk unhealthy. It cannot be doubted, that every departure from a sound physiological state in the nurse, is attended with some variation in the quality of her milk; but to ascribe all the effects of different kinds of food and drinks on her milk, to that cause, is to disregard the numerous facts, which observations on the lower animals have accumulated. Let us review some of these, beginning with those which relate to the milk and butter of the cow.

Dr. Young, in his Course of Experimental Agriculture, vol. 4, p. 370, states, that he had a cow fed totally on turnips, and that her cream tasted strong, and the butter was excessively bad.

Johnson (Anim. Chem., vol. 1, p. 138–9) states, that "all plants of the garlic kind, many of the umbelliferous, the horse mint, cabbages, turnips, and different kinds of autumnal leaves, give to the milk of the cow a peculiar flavor. Cows, continues he, fed on the leaves of maize, give an extremely mild and saccharine milk: those fed on the potatoe, an insipid milk."

Dr. Gardner adds, he has found, that after cows eat indigo, in the indigo fields, the cream of their milk is of a beautiful blue color. Cows that eat madder, he adds, give a milk that appears streaked with blood, and those which feed on the fruit of the cactus opuntia afford milk of a reddish color.

It was asserted by Professor Barton, in his Lectures, that all the plants of the class Tetradynamia impart peculiar qualities to the milk.

Another authority, of the highest credibility, is Parmentier, who holds the following language: "If the savor of milk," says he, "(independently of the peculiar nature of the animal,) be owing to the re-union of the different principles, that constitute this fluid; it is not less true, that these principles receive, on the part of vegetables, characters which are in some sort indelible. If plants contain, for example, mucus (mucilage) in abundance, the milk will give a great deal of caseous matter, (they both contain azote,) and its savor will be insipid or sweet. If, on the contrary, they are aromatic, the butter will be sapid from the affinity of the *esprit recteur* with the oily matter. In the same manner, the milk will be colored if the plants contain a coloring matter, soluble in one of the principles; and it will abound in whey or serum, if the plants contain much humidity. In short, all the products will be more fine, more solid, and more perfect, according to the tenuity of the oily, mucilaginous substances, and to the coriaceous, hard, and fibrous state of the plants, which concur in their formation. This being admitted, it is easy to see why the most perfect butter, and the most esteemed cheeses are made from milk of cattle nourished in meadows, where many odoriferous plants grow; and when these meadows have lost, by exsiccation, their perfume and their superabundant humidity, they give a less delicate and more firm butter; while cows furnished simply with the stalk and leaves of maize, give always a sweet milk and insipid and firm butter, on account of the insolubility of the saccharine body in the butter, of the absence of the aromatic part, and of the solidity of the vegetable. It will farther explain why the milk of the cow, fed with potatoe tops, which are more aqueous than maize, gives equally an insipid butter, but of a less firm consistence; why the cruciform plants communicate a strong taste to butter, while the whey is almost insipid; in short, why cows, that feed in moist places, give milk less rich than those feeding on high and open pastures. If, therefore, we wish to make the butter and cheese of cows fed in the first pasture, more perfect, some aromatic plants must be added to their common food; whilst succulent and inodorous plants, must be added to those nourished in the second; for good pastures depend as much on the soil and aspect, as on the variety of plants of which they are composed. To conclude, the foundation of the opinion of authors, who say that manipulation does every thing respecting the quality and the quantity of butter and cheese, and the pastures nothing, is totally overthrown by reason and experience; for the influence of vegetables on the nature and quantity of these two products is evident, as well as that of the processes employed to facilitate them."

3. *Of the Breath.*—An attention to the expired air, discloses to us, that many things taken at the table, often find their way, more or less undecomposed, into the blood of the pulmonary artery, and then go off with the aqueous vapor secreted by the lungs. The odor of onions and garlic has been observed by the whole world, and the aroma of whiskey and other ardent spirits, may at any time be perceived in the breath of one who has drunk to excess. In these cases the peculiar smell is not derived from portions of these different articles lodging about the teeth, for no detergence can remove it; the smell continues for several hours after the ingestion; and is as perceptible in the air expired through the nostrils, as in that which issues from the mouth.

4. *Of the Perspiration.*—We are assured by travelers, that the Icelanders who live mainly on fish and fish oil, have a perspiration which is exceedingly offensive, especially when they are crowded together in a warm room, and that it bears no little resemblance to the odor of the oil which they eat.

5. *Of the Urine.*—Dr. Johnson (An. Chem. Vol. 2, p. 366) asserts that asparagus, olives, garlick, onions, artichokes, horse radish, and some other vegetables, communicate to urine a peculiar and disagreeable smell.

Macquer, he informs us, declares that he has known persons, accustomed to head-ache and bad digestion, whose urine retained an evident odor of the food they had taken, such as coffee, roots of various sorts, fruits, pulse, and even soup and broths when made of indigestible meats.

Dr. Wollaston has shown that when birds are nourished solely upon plants, or non azotic substances, their urine scarcely contains any acid; while on the contrary it is very great, when they are nourished by substances containing azote, (Orfila Med. Chim. tom 2 p. 440.)

Dr. Magendie fed a dog on sugar till he died. The bladder was distended with urine, which Cheveruel found to possess all the characters belonging to that of herbivorous animals; that is, instead of being acid like that of the dog and other carnivorous animals, it was sensibly alkaline, not exhibiting any trace of uric or phosphoric acid. It was in short destitute of azote. The experiment was repeated twice with the same results. I may add that the bile in these cases afforded a considerable portion of picromel, a substance which contains no azote and is said to be peculiar to the bile of herbivorous animals.

6. *Of the Blood.*—As the milk, pulmonary vapor, perspiration, and urine are derived from the blood, it follows that the foreign matters which I have enumerated, as being sometimes present in them, must in all cases have been previously in that fluid. When there, they are dissolved in its water. It would, obviously, be more difficult to detect them in the blood than in the secretions, for in the latter they are more concentrated, and they are accumulated in the channels by which they are to be conveyed out of the sanguiferous system. They are of course not decomposed, in the blood vessels, for if so, there would be no necessity for depurating the system of them; and, moreover, if once decomposed, they could not, being organic compounds, ever be recomposed, in the excretions, which are no longer under the laws of organic but inorganic chemistry.

II. Of the modifications of the flesh and other solids in animals from changes in their food and drinks.

The flesh of the sheep, is well known to be remarkably influenced by its food. Wet pastures, with rank vegetation, give to mutton a bad quality; while dry hills, abounding in aromatic plants, render it delicious.

The beef of the ox that has run at large and subsisted only on wild grasses, is very different from that of the same animal that has been kept in the stable. That fed on oil cake, which will fatten them sooner than any other food, is bad.

The flesh of the hog is especially influenced by its food. Doctor Pearson (Practical Synopsis, p. 28) observes, "In Corsica where the hogs feed on chesnuts, and in Persia where they are often fed on dates, their flesh is peculiarly good, and the same remark is true," continues he "of some tropical countries, where they eat the sugar cane."

In this country, the flesh and fat of hogs which subsist and are fatted upon acorns and beech nuts, is strikingly different from that of those fed on Indian corn. It has a peculiar smell and taste, and is much softer than corn-fed pork; approaching, indeed, very much to the nature and properties of bear's flesh and oil, which are formed from the same materials; and, indicating to us that the digestive and nutrient organs of animals, as different as the bear and the hog, when supplied with the same aliment, produce from it parts not very unlike each other. It is well known, that if, when the hog has been fatted on acorns and beech nuts, it is put for a few weeks on Indian corn, the quality of its fat and flesh is materially changed.

The pork that is produced from the sediment of a distillery, has a different taste and smell from that produced from corn.

When the hog has subsisted in a great degree upon shell fish, which it does in the neighborhood of bays and harbors, its flesh has a peculiar taste; and, if I have not been misinformed, when it eats cotton seeds, the pork it affords is extremely unpleasant.

The flesh of several species of water-fowl, particularly the brentgoose, puffin, solan goose and gull, has an offensive, fishy flavor and odor, from those birds subsisting on fish. Pearson, p. 31–32.

Mr. Hunter fed two ducks for a month, one on barley, the other with sprats. They were killed at the same time. When they were dressed, the latter tasted so strong of fish that it could scarcely be eaten.—Ibid.

In the winter season, when, from a scarcity of their common food, several species of thrush (turdus) are compelled to subsist on berries of the misseltoe, ivy, holly, and spindle tree, (Euonymus Europœus,) their flesh becomes bitter, and acquires a purgative quality.—Ibid, 33.

The anas ferina, or canvas-back duck, feeds on the roots of the Valisneria Americana, in the Chesapeake bay, and this renders its flesh the most exquisite dainty within the reach of the American epicure.

The deer of America, sometimes feed on the berries of the Kalmia latifolia, and thence their flesh, it is asserted by Professor Barton, acquires purgative properties.

The flesh of wild pigeons, which have eaten poke berries, (Phyy. decand.,) it is said, by the same naturalist, is purgative.

Haller asserts, that the smell of garlic is perceptible in the flesh of the peacock, that has fed on it.

III. Of modifications of the solids and fluids of animals, by subjecting them to articles of food, which they do not naturally eat, or by confining them to a single substance.

If the digestive apparatus possesses the power of forming the same kind of chyle, out of every nutritious substance that is not poisonous, it must be able to supply those principles of our composition that are essential to it, and which may not be present in the aliment used. On this subject, experiments have not been numerous.

Azote is the characteristic ingredient of animals; but whether this be a simple body, we do not certainly know; the alkalies and water seem to act like simple bodies, and till lately were considered as such. They are compounds; and azote, may be, has indeed been suspected of being, also a compound. But, even, animals that are most strictly herbivorous, take in no small quantity of azote, in the innumerable variety and number of insects that adhere to the vegetables on which they subsist; in the myriads of animalcules which inhabit the waters they drink; in the atmospheric air that is blended with their food and water; and in the albumen, gluten and mucilage, and several other principles of plants that contain azote, to say nothing of the probability, that a portion of azote is absorbed in respiration.

The point can only be settled by causing an animal accustomed to a mixed diet, to subsist on one destitute of azote; keeping it at the same time upon distilled water.

This has been done by Magendie, (see Elements of Physiology, p. 376,) who fed a dog on pure white sugar, composed of hydrogen, oxygen, and carbon, and gave him no drink but distilled water. For seven or eight days he appeared to be very well, was sprightly, ate with avidity, and drank as usual. He began to grow thin the second week, although his appetite was good, and he ate six or eight ounces of sugar every twenty-four hours. His alvine excretions were neither frequent nor copious, but his urine was in sufficient abundance. The emaciation increased in the third week, the strength diminished, the animal lost its spirits, and its appetite became less. At this period there happened, first upon one eye, and then upon the other, a small ulcer, in the centre of the transparent cornea; it augmented rapidly, and in the end of a few days it was about a line in diameter, its depth increasing in the same ratio; the cornea soon became perforated, and the humors of the eye flowed out. This singular phenomenon was accompanied by an abundant secretion from the glands about the eyelids. In the meantime, the emaciation continued to increase, and the strength diminished daily; and though the animal, every 24 hours, ate three or four ounces of sugar, its weakness became so great, that it could neither chew nor swallow, and it expired on the 22d day of the experiment. I examined the body, continues the author, with every possible precaution, and there was no fat to be found. The muscles were reduced more than five-sixths of their ordinary volume, the stomach and intestines were much diminished in size and strongly contracted. The gall and urinary bladders were distended with their peculiar fluids." These fluids, I have already stated, approached very nearly to those of herbivorous animals, in having no azote, nor, according to Magendie, was there much of that substance found in the excrements.

Two other animals, subjected to the same diet, died with the same symptoms, and exhibited on dissection, the same appearances.

Two other animals of the same kind, were then fed on olive oil and distilled water. They did not experience an ulceration of the cornea, but had all the other symptoms, and after death exhibited the same anatomical characters.

To several dogs he then gave gum arabic. It produced the same results as the sugar and oil, except that an ulceration of the cornea did not occur as with the former substance.

Finally, he fed a dog exclusively on butter. He continued to eat it till twenty days before his death, which occurred on the thirty-sixth day. The right eye of this animal became ulcerated like those of the dog first mentioned. The other symptoms, appearances after death, and chemical composition of the fluids, were as in the first instance.

To satisfy himself that these different substances were digested, he fed several dogs with them, and in a few hours afterwards opened them. He found both chyme and chyle. The quality of the latter I have already mentioned.

From these experiments it results, that an animal accustomed to a mixed diet, when reduced to one purely vegetable, that is, one containing no azote, cannot exist; and that even the absorption from its own solids, and from the atmosphere, were not sufficient to supply azote for the secretions in which that principle is usually present.

The causes and symptoms of the disease denominated scurvy, may here be referred to with propriety. Nothing is more certain than that a diet of salt meat, without any fresh vegetables, continued for a long time, is capable of producing this disease; the phenomena of which indicate astonishing changes, both in the solids and fluids. In the course of the disease there is a general hæmorrhagic disposition. In this state, a diet of fresh and ascescent vegetables will effect an immediate cure. Every circumstance to my mind indicates, that the disease is in a great degree produced by the want of the proper kind of materials for maintaining the usual composition of the blood and solids, and supplying the waste of the body; and that it is cured, not by the action upon the stomach of the simple remedies of which I have spoken, but by the introduction of some part of them into the system with the chyle.

In Diabetes mellites Dr. Rollo found, that the secretion of sugar by the kidneys, was immediately stopped by a diet of animal food. In some of his patients who continued it for a great length of time, there was a secretion of oil on the surface of the body, fœtid breath, saltish taste, languor, and other symptoms which he considered indicative of approaching scurvy.

Finally, Dr. Stark made a number of experiments to determine the effects of different articles of food when persevered in for some time. His first experiment was with bread and water; which produced no very marked effects. To this succeeded a diet of the same kind with sugar. After he had continued this diet for about a fortnight, he perceived small ulcers over the inside of his cheeks. The gums were swelled, red, and bled when pressed with the finger; and the right nostril was internally red and purple, and very painful. After this, small purple streaks appeared on his left shoulder. His appetite now failed, and he could not taste sugar, which induced him to have recourse to wine and meat. During the continuance of this diet, he had in general every day three or four liquid alvine evacuations, containing some clear gelatinous matter, and he felt but little pain or flatulence in his bowels.—(*Edin. Med. Coment. for* 1788, *vol.* 7.)

General Conclusions.

1. Both the solids and fluids of the body are affected in their composition and qualities, by the food on which the animal subsists.

2. The system, at all times, makes an effort for the production of chyle and blood of the same quality, in each species; and when the food thrown into the system, is too unlike the composition of our bodies, disease, and even death itself may be the consequence; and in these cases the morbid condition exists primarily in the fluids.

3. This involves no paradox, for the fluids existed before the solids; and every thing taken in to supply the waste of the system, becomes a part of the fluids, before it enters into the composition of the solids.

4. The fluids, as far as we can observe, and I profess to go no farther than we can travel by the light of manifest phenomena, are equally concerned with the solids, in the great function of nutrition. Without both solids and fluids, there could be no circulation, and without circulation there could be no nutrition. Solids are the mechanical, fluids the chemical agents of the vital power, and they are equally necessary.

5. The question of the relation of alimentary substances, to the living body, is one of pure observation. We have no principle by which we can decide it *a priori;* for the living body is unique in nature, and governed by its own peculiar laws. One of these laws is, that the animal shall act upon and change the constitution of surrounding objects;—another, that it shall be acted upon and its physical condition altered by them. Perfect assimilation is the ultimate term of its influence upon *them.* Death, by the termination of all the functions, is the maximum of *their* influence over *it.* Among them, there are some on which it can produce no change: others which it invariably brings to its own condition. It acts upon them, and they react. It places them on its procrustus' bed, but they struggle against its amputations; and many of them, in virtue of *their* vis conservatrix, enter the living system but partially changed, in defiance of *its* vis conservatrix. Each strives to maintain its identity, and within certain limits each succeeds:—to a certain extent, they both fail. Thus the animal seeks to preserve its actions, sensations, functions, and the *composition of its solids and fluids*, in a *uniform*

state; but it fails, for it can accomplish *nothing perfectly.* The functions, and the composition of the organs that perform them, are *both* liable to variations; and, as we might have expected, had we looked at the matter without prejudice, are subjected to the same law, for they are governed by the same power.

I consider, then, the alleged absolute dominion of the organs of nutrition and assimilation, over alimentary matters, as a perfect anomaly in the plan of living nature; and the pretended uniformity of the flesh and blood, of the same animal under wide diversities of diet and drinks, as a philosophical chimera, unsustained by facts, and unworthy of modern science.

